# Air invasion into three-dimensional foam induces viscous fingering instabilities

**DOI:** 10.1038/s41598-024-53566-3

**Published:** 2024-02-05

**Authors:** Young H. Lee, Jingyi Wang, Ranjani Kannaiyan, Yi Su, Ian D. Gates

**Affiliations:** Department of Chemical and Petroleum Engineering, University of Calgary, Calgary, AB Canada

**Keywords:** Viscous fingering, Hele-Shaw, Foam displacement, Air injection, Foam flow, Fluids, Wetting

## Abstract

We conducted an experimental investigation to examine the immiscible radial displacement flows of air invading three-dimensional foam in a Hele-Shaw cell. Our study successfully identified three distinct flow regimes. In the initial regime, characterized by relatively low fingertip velocities, the foam underwent a slow displacement through plug flow. During this process, the three-phase contact lines slipped at the cell walls. Notably, we discovered that the air injection pressure exhibited a proportional relationship with the power of the fingertip velocity. This relationship demonstrated excellent agreement with a power law, where the exponent was determined to be 2/3. Transitioning to the second regime, we observed relatively high velocities, resulting in the displacement of the foam as a plug within single layers of foam bubbles. The movement of these bubbles near the cell walls was notably slower. Similar to the first regime, the behavior in this regime also adhered to a power law. In the third regime, which manifested at higher air injection pressures, the development of air fingers occurred through narrow channels. These channels had the potential to isolate the air fingers as they underwent a process of "healing." Furthermore, our results unveiled a significant finding that the width of the air fingers exhibited a continuous scaling with the air injection pressure, irrespective of the flow regimes being observed.

## Introduction

Instabilities of the interface between two phases flowing through porous media have been extensively studied due to their prevalence in various industrial processes. In situations where a lower-viscosity fluid displaces a higher-viscosity fluid within a porous medium, the interface between the two fluids undergoes deformation caused by instability. Under the instability, the deformation of the interface grows and can harm displacement process performance as well as eventual product quality that rely on uniform properties. The first experimental investigation of this instability, known as viscous fingering instability, was conducted by Hill^1^. In his experiment, he displaced sugar liquors with water in a vertically packed column of charcoal and observed viscous fingering instability characterized by the formation of fingerlike patterns, where the displacing fluid penetrates the displaced fluid^2^. This phenomenon is observed in a wide range of applications, including filtration, fluidization, enhanced oil recovery (EOR) processes, groundwater infiltration, soil remediation, geothermal energy production, CO_2_ sequestration, production of hollow fiber membranes, and air-assisted injection molding^3–7^.

The existing literature on viscous fingering instabilities has predominantly focused on Newtonian fluids. However, experimental studies have revealed significant differences in the fingering patterns when non-Newtonian fluids are involved^8,9^. Nittmann et al. conducted rectilinear miscible displacement experiments in a Hele-Shaw cell, observing a fractal growth of viscous fingers when non-Newtonian polymer solutions were displaced by water^10^. Similarly, Daccord and Nittmann observed fractal viscous fingers in a radial Hele-Shaw cell when high-viscosity non-Newtonian polymer solutions were displaced by water^11^.

In our daily lives, the majority of fluids we encounter, such as shaving foam, glues, flour–water dough, mayonnaise, and paints, exhibit non-Newtonian behavior. These fluids often display multiple non-Newtonian properties simultaneously, including shear-thinning or shear-thickening, yield stress, and viscoelastic effects^6,12^. As a result, understanding viscous fingering instabilities in non-Newtonian fluids becomes more complex and challenging^13^. Liquid-based foam is a non-Newtonian fluid composed of gas bubbles randomly dispersed in a liquid where typically, the volume fraction of gas far exceeds the volume fraction of liquid^14,15^. Foams are used in many household and industrial products where a liquid foam is allowed to solidify to form a solid form, e.g., polyurethane foams. Foams exhibits flow properties arising not only from the viscosity of the liquid and gas phases but also the interfacial forces at the interfaces and Plateau borders^16^. At particular conditions, foam acts elastically like a solid at low shear stress but flows like a liquid at high shear stress^14^. At other conditions, foam can also flow like a shear-thinning fluid^15^. When a foam undergoes deformation and flow, its internal resistance to flow arises from viscous forces within the phases (liquid, gas) and between the phases (liquid, gas, and solid bounding surfaces) but also the energy required to stretch or deform interfaces which will tend to evolve to a configuration where the overall surface energy is minimized^15,17–19^.

There are a few studies on the displacement of liquid foam by gas in a Hele-Shaw cell. Injecting gas at a continuous flow rate or constant pressure into a body of liquid foam in a Hele-Shaw cell leads to finger-like gas/foam interfaces^20^. This behavior is the well-known Saffman-Taylor instability because viscous foam is pushed by a less viscous gas in a porous medium^2,21^. Hilgenfeldt et al. examined foam dynamics of a quasi-two-dimensional liquid foam in a rectangular Hele-Shaw cell by injecting air at constant pressure at one end of the cell^22^. At very low pressures, the entire foam was uniformly displaced. It was observed for pressures larger than a critical value, fngers of air propagate through the foam through the rearrangement of bubbles where bubble neighbors are swapped (a T1 process). They also found a foam yielding to the injected air by breaking the thin films between bubbles.

Arif et al.^23^ explored crack propagation in dry, quasi-two-dimensional aqueous soap foam in a rectangular Hele-Shaw cell by injecting air at constant pressure. They observed a finger-like propagation of ductile fracturing under a T1 process around the fingertip at moderate speed and a propagation of brittle crack by breaking films at much higher speed. They also found that there is an upper limit to the ductile crack speed and a lower limit to the brittle crack speed. The fracture of a quasi-two-dimensional aqueous foam under an applied driving pressure was also investigated through simulations using a network modelling approach^24^. Salem et al.^21^ investigated the response of a quasi-two-dimensional liquid foam to air injection in a radial Hele-Shaw cell. They identified swelling regime with fingering patterns and high-speed regime with a series of film ruptures. They examined the dependence among the swelling rate (increasing rate of fingering area), injection pressure, and other parameters. In a subsequent study, Salem et al. examined influence of surfactants on the response of a quasi-two-dimensional liquid foam in a radial Hele-Shaw cell^25^.

Yanagisawa and Kurita examined the collective relaxation response of a two-dimensional wet foam to a disturbance^26^. A perturbation was made by injecting a constant amount of additional solution from the outside. They observed two sharp peaks in the movements of foam bubbles during the relaxing process. The first peak movement was just a T1 event. The second peak movement was a localized collective movement of bubbles occurring at the same time. They focused on the second peak collective movement and defined the relaxation time as the duration of the second peak movement. They found that the relaxation time and correlation lengths for the collective movements increase with $\phi$. They also found that monodisperse foams showed slip-like motion in one direction while polydisperse foams showed random motion. Park and Durian investigated pattern formation in the immiscible radial displacement of Gillette shaving foam (Foamy regular) in Hele-Shaw cell^27^. In their experiments, nitrogen gas at constant pressures between 1 and 20 kPa was injected into Hele-Shaw cell to displace foam. They found that the finger width scales with the driving pressure, and the proportionality constant increases with the gap thickness divided by bubble diameter. However, the Hele-Shaw cell was not rigid enough to inject nitrogen at pressures higher than 20 kPa.

Lindner et al. studied the instability for a polymer gel and Gillette shaving foam (Foamy regular) in a rectilinear Hele-Shaw cell^12^. They observed very branched fingers in the gel, but the results for foams were very different because the foam slipped at the wall. The complexity of the displacement of foam arises from its multi-phase nature—a displacement could either push the foam structure through the space as a whole or potentially split an interface leading to a bubble ‘poking’ its way along bubble interfaces with little displacement of the bulk of the foam. Sliding of bubbles on the interfaces could also occur.

In this work, we investigate the instabilities associated with the immiscible radial displacement flows of air invading foam in a radial Hele-Shaw cell achieving pressures higher than that of Park and Durian^27^. We study radial displacement flow of three-dimensional foam which contains many bubbles in the thickness direction, while the quasi-two-dimensional foam in the previous studies consists of bubbles squeezed between two plates. In addition, we cover higher air injection pressures using a rigid Hele-Shaw cell to avoid flexing of the glass plates. We provide the first detailed investigation of the fingertip velocity, number of fingers, and other quantitative values on fingering patterns as a function of the air injection pressure for the radial viscous fingering of three-dimensional foam.

## Experiments

**Figure 1:**
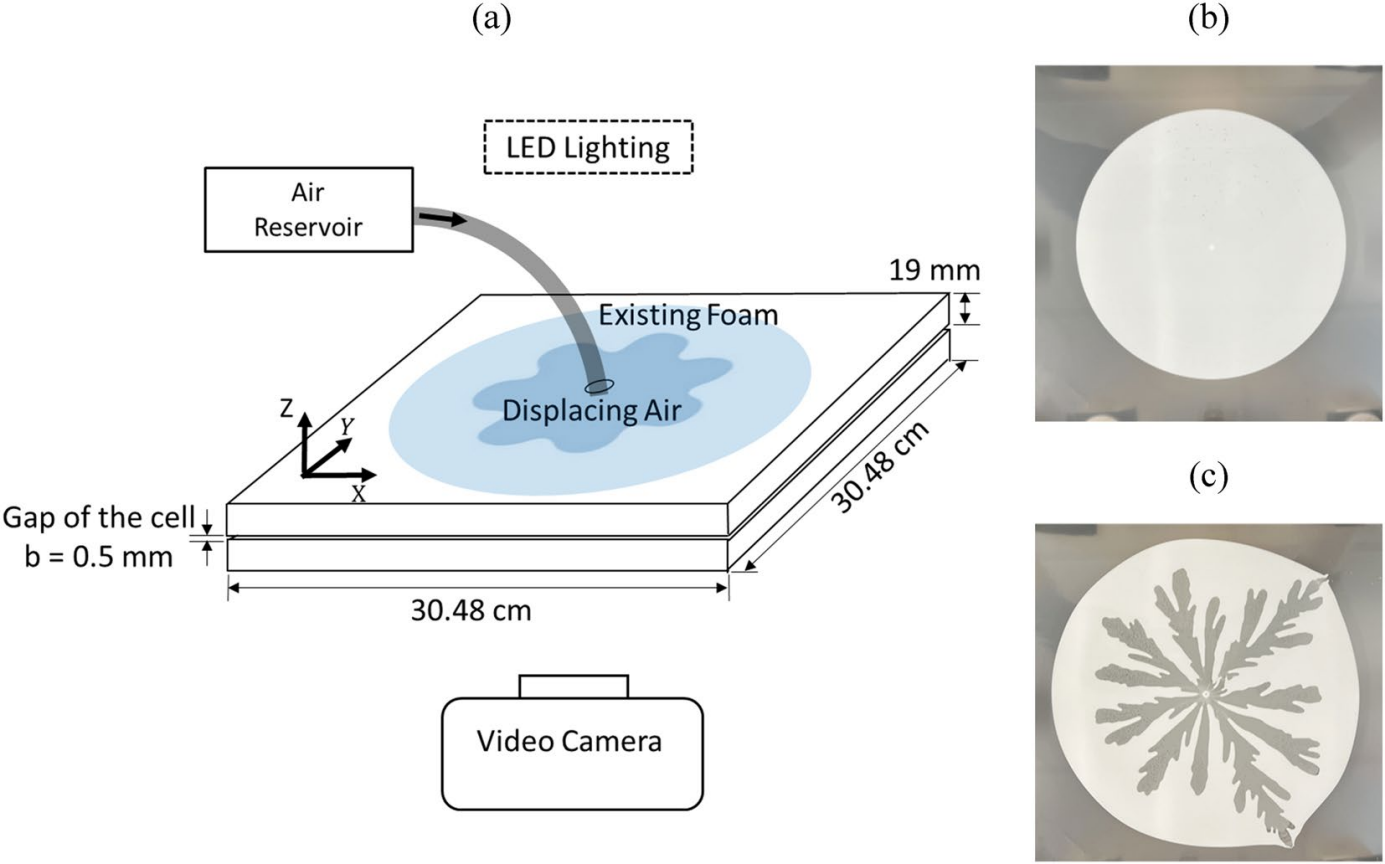
(**a**) Schematic of the radial viscous fingering experimental setup for air invading foam. (**b**) Gillette Foamy Regular shaving foam slowly injected using a 60 cc syringe to a sample radius, ${\text{R}}=87.7\pm 0.5\mathrm{ mm}$, and (**c**) air is injected into the cell from a reservoir tank at constant pressure.

In the study, viscous fingering (VF) experiments were conducted by using a radial Hele-Shaw cell apparatus. The cell consisted of two square borosilicate glass plates (304.8 mm × 304.8 mm × 19 mm, SCHOTT Borofloat® 33), as depicted in Fig. 1a. To achieve a controlled gap thickness, the two plates were clamped together using Polyester Plastic shims of 0.508 mm (0.02 inches) thickness. These shims were placed symmetrically at eight positions between the plates. An injection hole with a diameter of 3.175 mm (0.125 inches) was positioned at the center of the top plate. The accuracy of the gap thickness was verified by comparing the volume of injected water with the area occupied by the water multiplied by the gap thickness. A commercial shaving foam, Gillette Foamy Regular, was initially placed within the gap in the Hele-Shaw cell. The shaving foam was slowly injected to the Hele-Shaw cell using a 60 cc syringe until it reached a radius, $R=87.7\pm 0.5 {\text{mm}}$.

For the displacement process, constant pressure air injection was carried out into the Hele-Shaw cell through the upper plate. This was achieved by utilizing a 15-gallon (56.8 L) reservoir tank at the desired pressure. To ensure the reliability of the experiments, a minimum of three experiments were performed under each set of conditions.

To enhance wettability and ensure uniformity, the inner surfaces of the Hele-Shaw cell were meticulously polished using a random orbital polisher equipped with a fine steel wool pad (Grade #00). Additionally, a light diffusing film was affixed to the top of the Hele-Shaw cell, placed beneath the LED lighting. This arrangement aimed to improve the visibility of the viscous fingering (VF) patterns during the experiments. Recording the experimental observations was accomplished using a vertically positioned video camera (GoPro HERO 10 Black) operating at a rate of 240 frames per second (time interval between each frame is ~ 4 ms). The camera was placed below the cell, serving the purpose of concealing the injection tube and maintaining a consistent distance from the cell throughout the recording process.

The Gillette Foamy Regular shaving foam has water, steric acid, triethanolamine, palmitic acid, isobutane, laureth-23, sodium lauryl sulfate, fragrance/parfum, and propane as its ingredients, and it produces a gas–liquid foam with $92\pm 1\%$ hydrocarbon gas bubbles by volume^14^. The bubbles have average diameter equal to about $30 \mathrm{\mu m}$ and standard deviation of about $14 \mathrm{\mu m}$ up to ~ 20 min which then increases due to coarsening^14^. The gap thickness of the Hele-Shaw cell is about 17 times the average bubble diameter. As a result, the shaving foam in this study is polydisperse three-dimensional. The surface tension of the shaving foam solution was determined as $\gamma =24.1\pm 0.1 {\text{mN}}/{\text{m}}$ at 20 °C measured by using a Wihelmy plate^28,29^. The shaving foam solution (the liquid phase of the foam) is a Newtonian fluid, and its viscosity (measured using a DHR-2 rheometer, TA Instruments Inc.) is $\mu =1.845 {\text{cP}}$ at 21 °C^29^. A three-dimensional foam is “dry foam” if the liquid volume fraction $\phi <0.05$, it is “wet foam” if $\phi >0.15$, and it is “bubbly liquid” if $\phi >0.36$^30^. The bubbles in “dry foam” are polyhedrons with very thin films, and bubbles in “wet foam” are round. The shaving foam used in this study has the liquid volume fraction of $\phi =0.08\pm 0.01$, and it lies between dry foam and wet foam. As a result, it is expected that the shaving foam bubbles are polyhedrons with thicker films and more roundness than dry foam bubbles. According to the study on collective relaxation dynamics in a two-dimensional foam, a wet foam with $\phi =0.09$ showed simultaneous collective rearrangement of bubbles while separated T1 events propagated in dry foams^26,31^. This suggests that the shaving foam with $\phi =0.08\pm 0.01$ can be considered as a wet foam.

To quantitatively assess the instability of the interfaces, the effective number of fingers^32^, finger area density^33–35^, representative finger width density^35^, and representative fingertip velocity are examined. The effective number of fingers is calculated from the Fourier spectrum of the radial interface position. More specifically, the interface is rendered as a vector of points, ***X***, of a radius versus angle, and its discrete Fourier transform is determined, given by $F(k)$ = fft(***X***) where the Matlab function fft is used, $F(k)$ is the discrete Fourier transform, and $k$ is the wave number. For X of length n, these transforms are defined as follows:

1 $$F\left(k\right)={\sum }_{j=1}^{n}X\left(j\right){W}_{n}^{(j-1)(k-1)}$$

where ${W}_{m}={e}^{-\frac{2\pi j}{m}}$. This give the units of $F(k)$ being the same as that of $X(k)$. The amplitude spectrum is given by $\left|F(k)\right|$. The effective number of fingers, ${k}_{{\text{eff}}}$, is then determined from:

2 $${k}_{eff}=\frac{\sum_{k}{\left|F(k)\right|}^{2}k}{\sum_{k}{\left|F(k)\right|}^{2}}$$

The finger area density, FAD, is defined as the ratio between overall area enclosed by any fingers (all the way back to the inlet) and $\pi {{r}_{tip}}^{2}$, where ${r}_{tip}$ is the radius of the point on the interface farthest from the center of the domain^33,36^. A FAD value of 1 is the case of a perfectly stable circular interface; the lower the value of FAD, the more complex is the fingering structure. The average finger width density ${d}_{w}(r)$ for a given $r$ is obtained as $\overline{w }/2\pi r$ where $\overline{w }$ is the average finger width^35^. The representative finger width density for the entire pattern, ${\overline{d} }_{w}$, is the average of ${d}_{w}(r)$ between ${r}_{base}$ and ${r}_{tip}$. The radius of the point on the interface closest from the center of the domain is ${r}_{base}$. The average finger width $\overline{w }$ is the quotient of the total widths of the fingers in a circle of radius $r$ and the number of fingers encountered in the circle. Multiple runs were conducted at each condition to ensure repeatability. The quantitative values, ${k}_{eff}$, ${\overline{d} }_{w}$, and FAD are determined from the fingering images when the fingertip radius reaches 60 mm. Figure 5 (in the Results and Discussion section below) provides an image illustrating the dimensions used in the analysis. The recorded video frames were processed in MATLAB to identify interfaces and obtain various quantitative values for fingering patterns.

## Results and discussion

**Figure 2:**
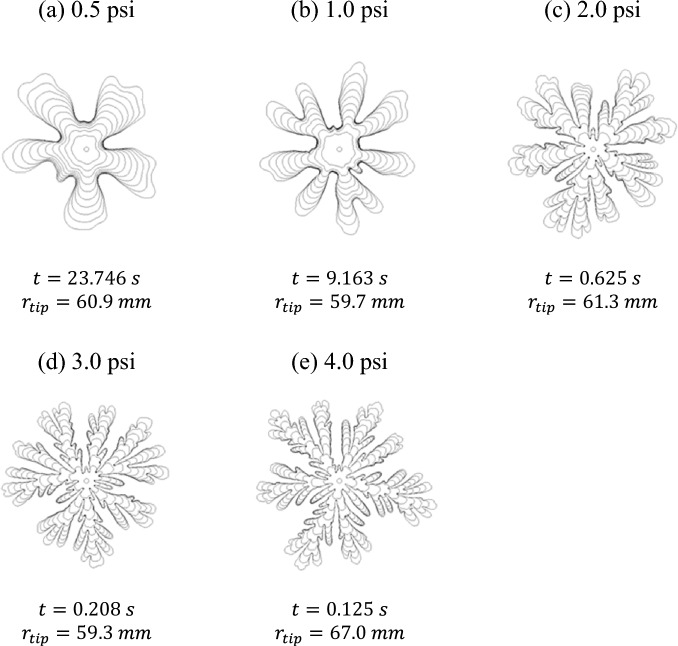
Time sequences of the viscous fingering interface at different air injection pressures (**a**) 0.5 psi, $\Delta {\text{t}}=2.371\mathrm{ s}$, (**b**) 1.0 psi, $\Delta {\text{t}}=0.913\mathrm{ s}$, (**c**) 2.0 psi, $\Delta {\text{t}}=0.063\mathrm{ s}$, (**d**) 3.0 psi, $\Delta {\text{t}}=0.021\mathrm{ s}$, and (**e**) 4.0 psi, $\Delta {\text{t}}=0.013\mathrm{ s}$. $\Delta {\text{t}}$ is the time intervals between interfaces shown in the images.

**Figure 3:**
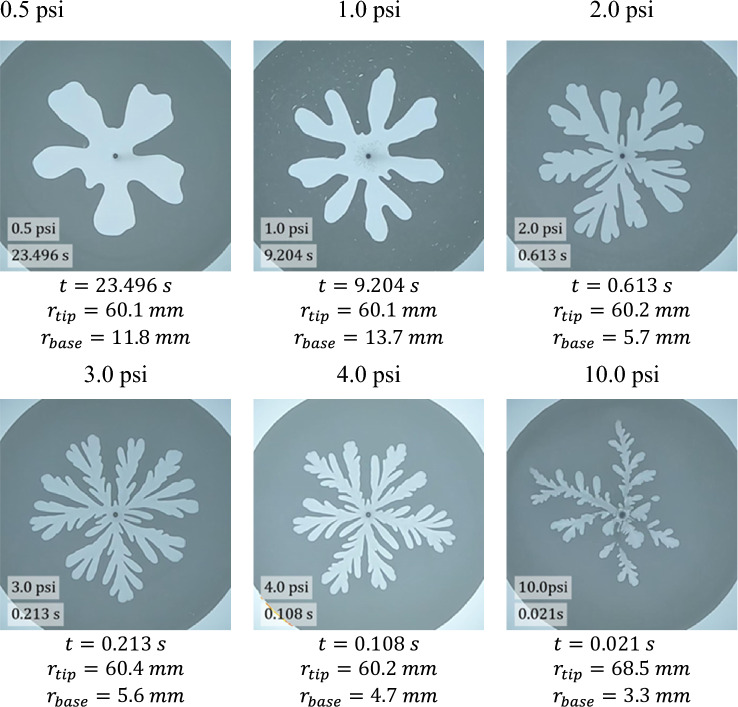
Viscous fingering images at different air injection pressures.

The fingering experiments were carried out by radially displacing the foam by injecting air at constant pressures between 0.5 and 10.0 psi (3.45 and 68.95 kPa) above atmosphere into the center of the Hele-Shaw cell. Figure 2 depicts time sequences of the air-foam interface at different air injection pressures of 0.5, 1.0, 2.0, 3.0, and 4.0 psi (3.45, 6.89, 13.79, 20.68, and 27.58 kPa), and air-foam images are presented in Fig. 3. At low pressures ($0.5\mathrm{ psi and }1.0\mathrm{ psi}$), the foam was displaced slowly by plug flow with apparent slip at the walls with the result of no residual foam bubbles remaining on the surfaces. This implies there is a thin wetting liquid layer between the surface and foam bubbles (Plateau borders touching the wall) that lubricates the foam flow within the gap^37,38^. It also implies that the cohesive strength of the foam arising from the interfacial tension is sufficiently strong so that as a unit, the foam remains intact and moves as a ‘solid’ body within the gap. The observations show that there is no relative motion of the bubbles relative to each other in the cross-gap direction. However, there is relative motion between the bubbles in the horizontal directions due to the expansion of the fingers both radially and laterally within the gap.

The evolution of the fingers at low pressures follows a typical immiscible radial viscous fingering pattern where the initial fingers spread and some of them split when the finger width becomes large enough as shown in Fig. 2^3,33^. The fingering pattern at 0.5 psi exhibits wide fingers and ‘gentle’ tip splitting is observed at the last interface displayed in the image. At 1.0 psi, the finger width becomes narrower and tip splitting starts at an earlier stage. A different viscous fingering pattern with even more narrower fingers and greater finger velocity is observed at 2.0 psi. At this pressure, the time for the fingertip to reach the same radial distance is about 15 times faster than that at 1.0 psi. The VF pattern also shows multiple levels of tip splitting and many ‘daughter’ branches. In addition, at 2.0 psi, a layer of foam bubbles remains on the Hele-Shaw cell walls where the foam is displaced by air. Park and Durian also observed that residues of foam bubbles are left behind at higher growth rates that they explored and that the residues are never more than two or three bubble diameters thick^27^. This means that slipping occurs mainly between bubble layers touching the wall and the next bubble layer towards the interior at higher pressures. In this case, the resistance forces for motion between the wall and the first layer of bubbles adjacent to the wall are larger than the deformation and flow resistance of the internal layers of bubbles and thus, a plug flow occurs within the internal layers of bubbles. A similar fingering evolution pattern is observed at 3.0 psi and 4.0 psi. However, the higher the pressure, the thinner is the finger width and greater the number of daughter branches.

**Figure 4:**
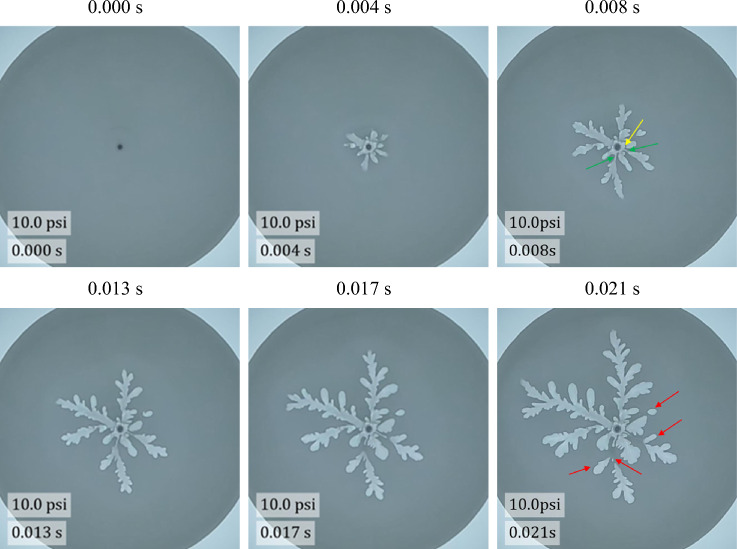
Evolution of viscous fingering at high driving pressure of 10.0 psi. Narrow channels are marked using a green arrow. The yellow arrow marks the presumed location of a narrow channel that is still feeding a growing finger, despite there being no visible connection between the finger and the inlet within the plane of the figure. The invisible channel is presumably in a different plane somewhere within the interior of the gap, so is necessarily narrower than the thickness of the Hele-Shaw gap. The red arrows highlight isolated finger tips.

Another significantly different fingering evolution is observed at 10.0 psi as shown in Fig. 4. At this high pressure, some of the fingers evolve as more narrow channels which then fill larger air invaded zones downstream of the channel. This is due to the reduction of the gas velocity as the air moves radially outward leading to a widening of the fingers away from the injection point. The faster the gas velocity, the greater the shear amongst bubble layers, and as a result, the more narrow the finger emerging outwards. As the gas velocity drops, the lower the shear, and the more wide the finger. The observations reveal that the narrow channels can disappear leading to an isolated air zone in the foam. This means the foam bubbles behaved elastically within initial deformation (forming the narrow channels) that can subsequently ‘heal’ restoring an intact foam where the channel once existed. The evolution of the narrow channels and ‘healing’ of the channels is a dynamic behaviour that depends on where the air is invading the foam at a given point of time—for those channels that become starved of air, the foam ‘heals’ the channel and the air pocket downstream becomes isolated and the fingers receiving the air still receiving air extend. The observations also reveal that some channels are not visible in the image, which means they are in the middle of the foam layer within the gap. In addition, the number of ‘parent’ fingers is lower than that at lower injection pressure but each one of the parent fingers has a greater number of ‘daughter’ fingers emitting from the ‘parent’ fingers. Isolated air pockets appear to form either within the entire gap or partially fill the gap of the Hele-Shaw cell.

**Figure 5:**
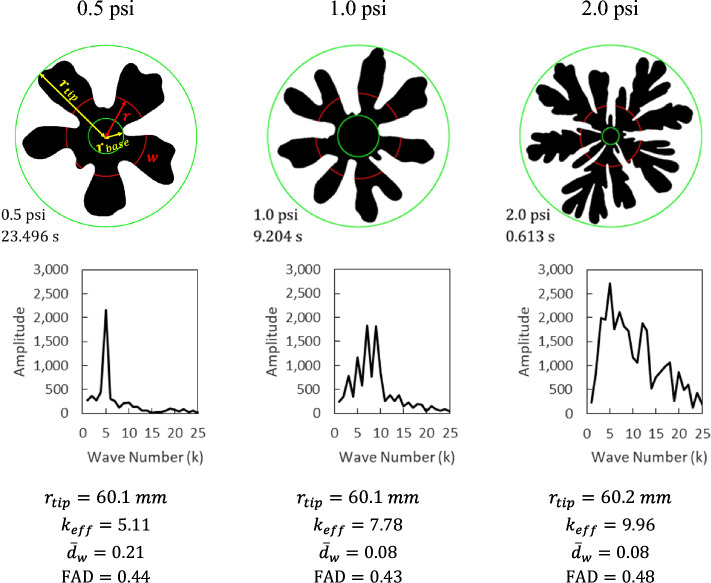
Viscous fingering image analysis, Fourier transform spectra of the interfaces, and calculated quantitative values for different air injection pressures.

Figure 5 illustrates how the effective number of fingers, the finger area density, the representative finger width density, and representative fingertip velocity are obtained using fingering patterns at air injection pressures of 0.5, 1.0, and 2.0 psi. As shown in Fig. 5, there is only one dominant wave number at ~ 5 with the largest amplitude for the fingering pattern at 0.5 psi, and the effective number of fingers is determined as ${k}_{eff}=5.11$. In the case of the fingering pattern at 1.0 psi, wave numbers at ~ 7 and ~ 9 are dominant resulting in ${k}_{eff}=7.78$. The Fourier spectrum at 2.0 psi represents five main fingering groups and many side branches. The effective number of fingers at 2,0 psi is ${k}_{eff}=9.96$ which is 2.18 times greater than that at 1.0 psi.

**Figure 6:**
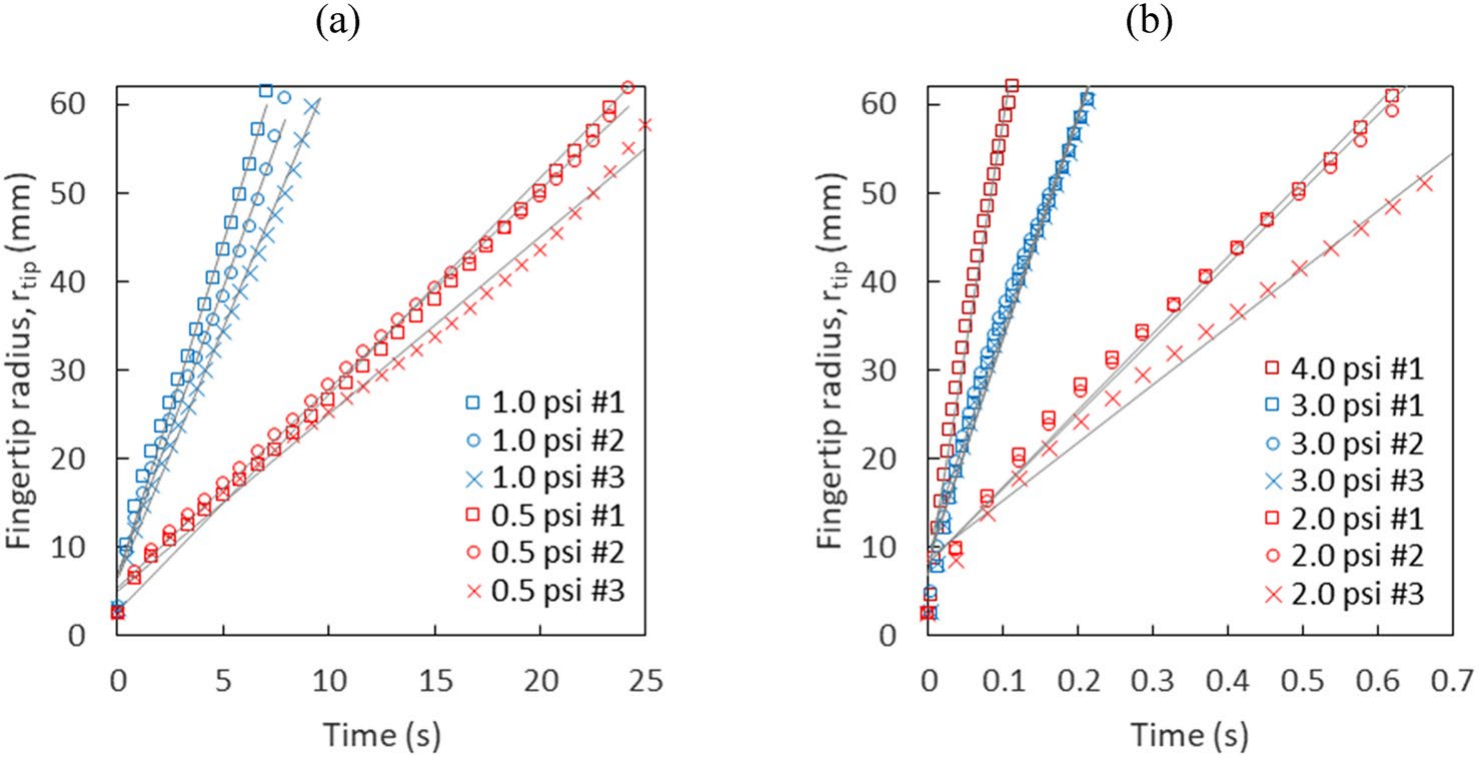
Fingertip radius ${{\text{r}}}_{{\text{tip}}}$ as a function of time for repeated experiments at (**a**) low and (**b**) high injection pressures. Fitting lines for each experiment are shown as gray lines.

**Figure 7:**
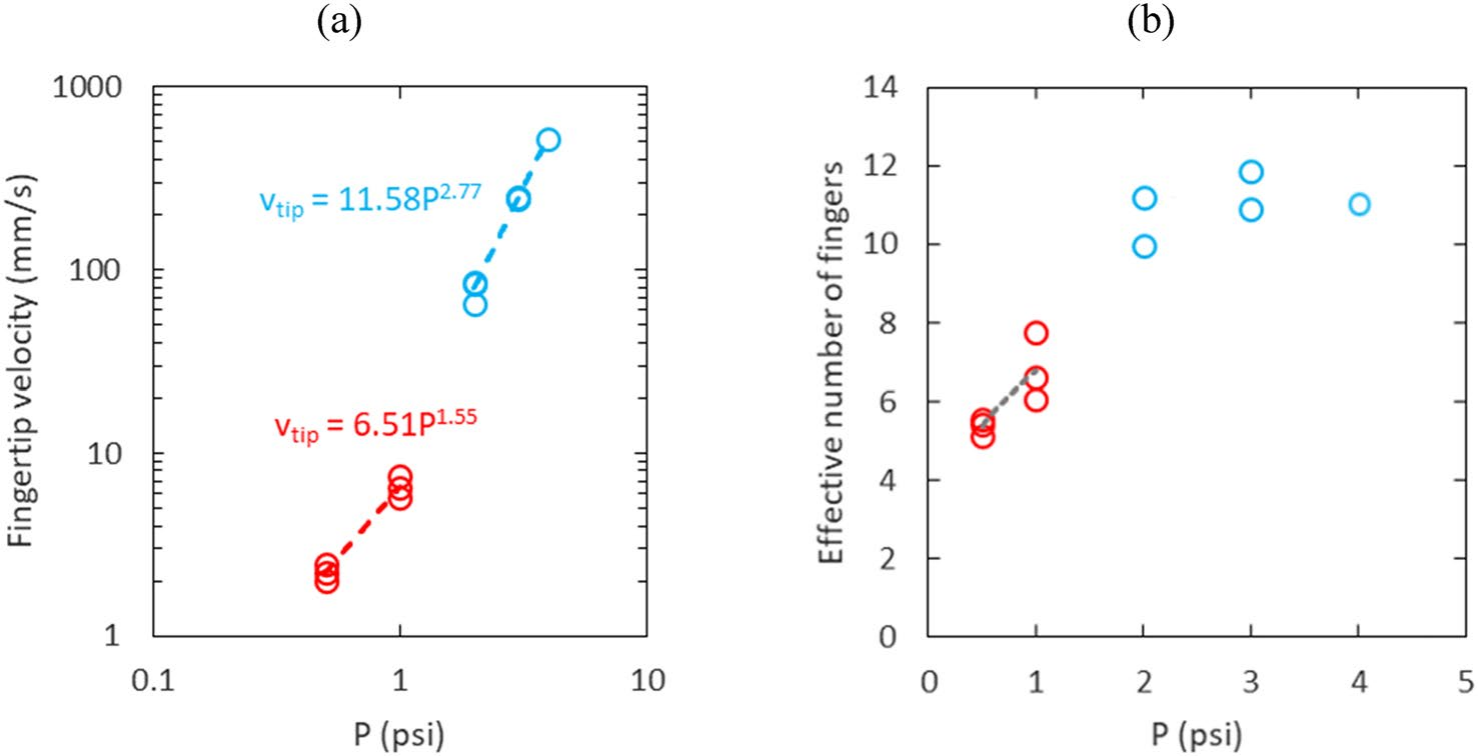
(**a**) Fingertip velocity as a function of air injection pressure and (**b**) effective number of fingers versus air injection pressure*.*

The representative fingertip velocity ${v}_{tip}$ is determined by fitting a line to the time series of the fingertip radius ${r}_{tip}$ as shown in Fig. 6. The fingertip velocities determined from the fitting lines are plotted as a function of pressure on a log–log scale in Fig. 7a. We identify three flow regimes according to how the foam is displaced. The three flow regimes with corresponding representative fingertip velocities ${v}_{tip}$ and capillary numbers^39^, ${Ca}^{*}=\mu {v}_{tip}/\gamma$, are listed in Table 1. Note, the velocity indicted in flow regime #3 exceeds 3 m/s.

**Table 1:** Representative fingertip velocities ${{\text{v}}}_{{\text{tip}}}$ and wall capillary numbers, ${{\text{Ca}}}^{*}=\upmu {{\text{v}}}_{{\text{tip}}}/\upgamma$, for the three flow regimes.

Flow regime	${v}_{tip} ({\text{mm}}/{\text{s}})$	${Ca}^{*}$
#1	$1.9<{v}_{tip}<7.5$	$1.5\times {10}^{-4}<{Ca}^{*}<5.7\times {10}^{-4}$
#2	$65.6<{v}_{tip}<516.6$	$5.0\times {10}^{-3}<{Ca}^{*}<4.0\times {10}^{-2}$
#3	3200	$2.5\times {10}^{-1}$

The liquid fraction and the size distribution can be the source of errors in each measurement shown in Fig. 6, which can be explained as follows. As a finger propagates, the existing foam bubbles are displaced and rearranged. The liquid fraction and the size distributions affect how the bubbles move and the time it takes for them to rearrange^26,31^. If there is a difference in the liquid fraction and the size distribution in each experiment, this will affect the evolution of fingering pattern. At lower pressures, we can expect more error because the growth of finger and the rearrangement of bubbles happen together slowly.

The pressure drop for bubbles moving with a velocity $V$ in a cylindrical or polygonal capillary is found to be proportional to a power of $V$^38,40,41^:

3 $$\Delta {\text{P}}\propto {V}^{\alpha } {\text{with}} \alpha =\frac{2}{3}.$$

This is consistent with other research in the literature where it was experimentally shown that the power law with the exponent $\alpha =2/3$ also applies to a layer of foam bubbles slipping in a rectilinear Hele-Shaw cell^42^. This relationship also means that the viscous friction between the sliding bubbles and confining wall is proportional to a power of $V$^15^. According to the study on the effects of surfactant type on the foam-wall viscous friction, the power law index $\alpha =1/2$ for foams with high surface modulus including Gillette shaving foams, and $\alpha =2/3$ for foams with low surface modulus^15^. As mentioned earlier, the relative motion of foam bubbles with respect to other bubbles in longitudinal directions was observed near the advancing fingertip. Therefore, the radial displacement flow of foam sliding on the walls involves both foam interface-cell wall friction and the viscous friction between foam bubbles.

It has been reported that the viscous stress in sheared foams is much higher for foams with high surface modulus than for foams with low surface modulus^43^. In this study, two power law exponents are identified by fitting two lines to the data points in Fig. 7a. The equations for the two lines are shown in Fig. 7a, and the slope is $1/\alpha$. The flow regime #1 where the foam is displaced slowly by plug flow slipping at the walls has the power law exponent $\alpha =0.65\approx 2/3$. This means that the radial displacement of foam by air in a Hele-Shaw cell is also in good agreement with the power law with the exponent $\alpha =2/3$. In flow regime #2 where the shaving foam is rapidly displaced leaving a layer of foam bubbles behind, the power law exponent is determined as $\alpha =0.36$. The layer of bubbles remaining on the plate indicates that there is some modification of structure in the two layers of bubbles adjacent to the boundary so that the viscous force between the two layers becomes less than the viscous force between the wall and bubbles. As a result, the foam bubbles slip on the first layer of bubbles adjacent to the boundary instead of slipping on the wall.

Figure 7b depicts the effective number of fingers versus air injection pressure. In the low velocity regime, the effective number of fingers increases from 5 to 7 due to tip splitting of main fingers as the pressure is raised from 0.5 to 1.0 psi. On the other hand, there is a noticeable increase of the effective number of fingers in the high velocity regime due to multiple levels of tip splitting and many daughter branches. Between 2.0 and 4.0 psi, the effective number of fingers ranges between 10 and 12.

**Figure 8:**
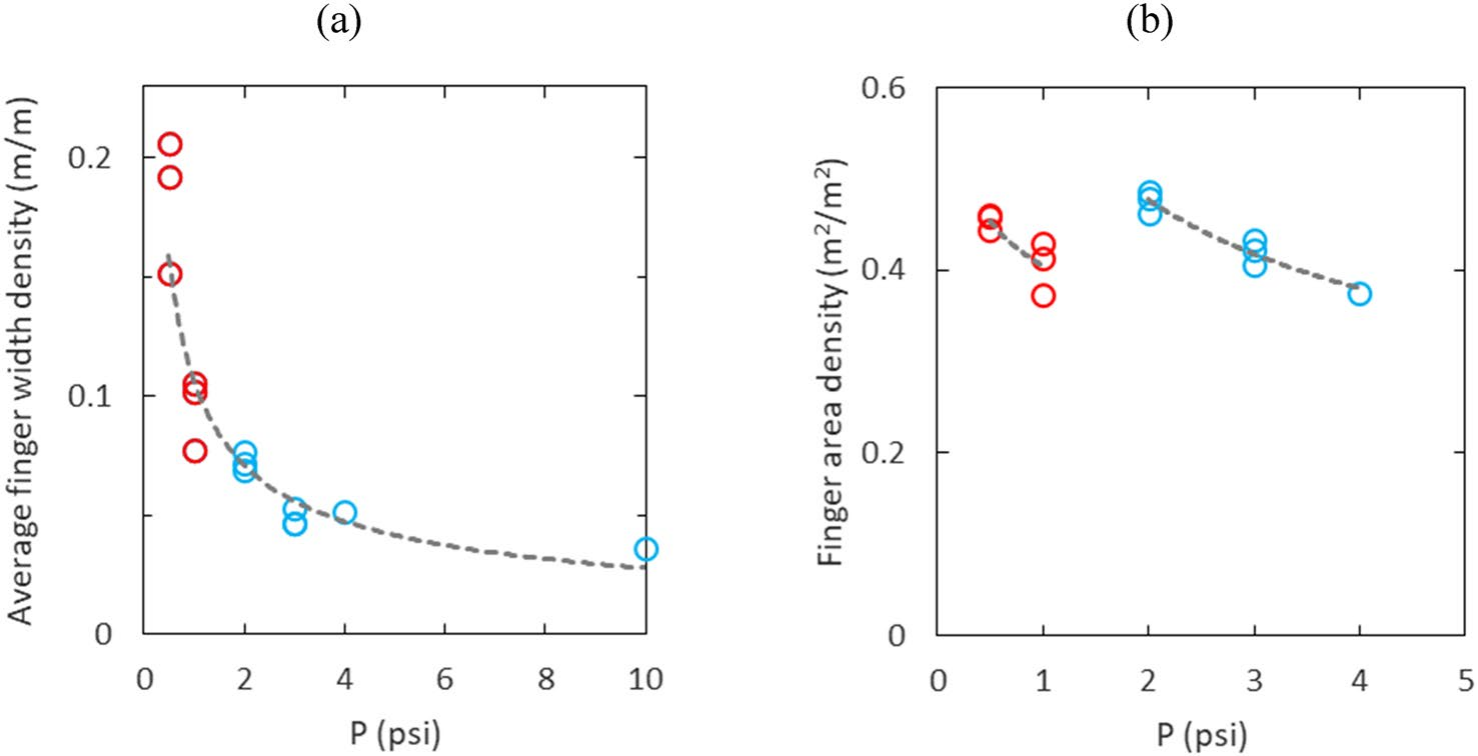
(**a**) Average finger width density (m/m) versus air injection pressure and (**b**) finger area density (${{\text{m}}}^{2}/{{\text{m}}}^{2}$) versus air injection pressure.

Figure 8a depicts the average finger width density versus air injection pressure. Unlike fingertip velocity, effective number of fingers, and finger area density, which show discontinuous or distinct patterns of change with respect to pressure in two flow regimes, the average finger width density continuously scales with air injection pressure regardless of the flow regimes as shown in Fig. 8a. The average finger width density scaling is ${\overline{d} }_{w}\propto {P}^{-0.58}$ from the fitting curve. This is consistent with the finger width scaling, $\lambda \propto {(P/R)}^{-0.5}$ where $\lambda$ is the finger width and $R$ is the sample radius, found by Park and Durian^27^. Figure 8b shows the finger area density versus air injection pressure. In both flow regimes, the finger area density starts at approximately 0.45 and decreases as the air injection pressure increases. It also shows noticeable discontinuity with respect to pressure between the two flow regimes. At 1.0 psi, the FAD is 0.4, and it increases to 0.48 at 2.0 psi as shown in Fig. 8b.

**Figure 9:**
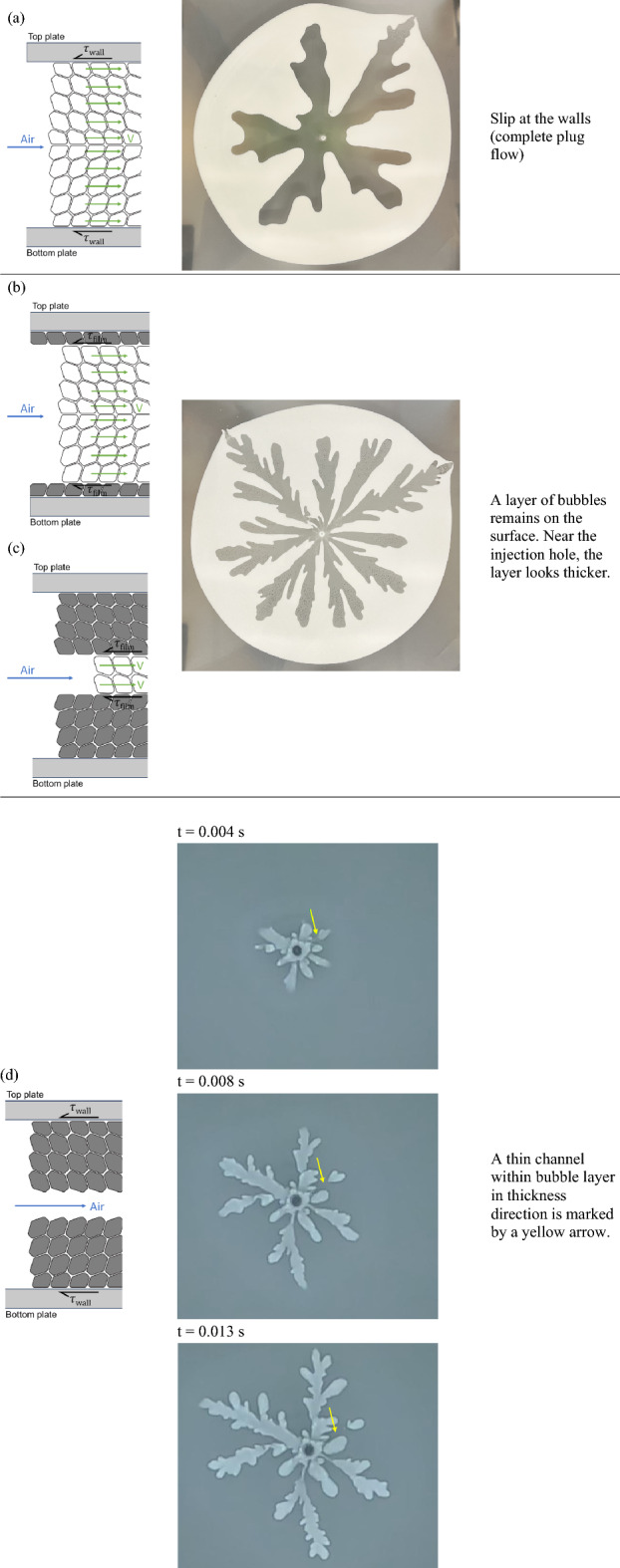
Schematics of foam displacement flows in Hele-Shaw cell in the cross-gap plane: (**a**) slip at the walls (complete plug flow), (**b**) slip at the first layer of bubbles adjacent to the boundary (internal plug flow), (**c**) slipping within internal layers of bubbles (internal plug flow), and (**d**) splitting of bubble interfaces (bubbles do not move).

Schematics of foam displacement flow in Hele-Shaw cell for the flow regimes #1, #2, and #3 are depicted in Fig. 9. As shown in Fig. 9a, it is hypothesized that the foam bubbles first respond elastically by deforming their shapes against the restoring force of surface tension and tilting in the direction of the shear stress from the wall as the air pressure pushes the foam bubbles in Hele-Shaw cell^15,41^. Next, the bubbles start sliding at the walls. We were able to confirm with the naked eye that no foam bubbles remained on the glass surface. This observation is consistent with that of Park and Durian^27^.

As the air pressure increases, the velocity at which the bubbles slide increases, and as a result, the foam-wall friction increases, causing the bubbles at the layers of bubbles adjacent to the boundary to deform more. As shown in Fig. 9b/c, we were able to confirm that a layer of bubbles remained on the glass surface. Sometimes, we noticed that some areas of the remaining layer are thicker than one bubble. Park and Durian also observed a layer of bubbles on the surface at higher flow rates^27^. They also mentioned that the layer is never more than two or three bubble diameters thick. It is hypothesized that the foam films between the two layers adjacent to the boundaries are flattened, resulting in reduced viscous friction between the two layers. When the viscous friction between two layers of bubbles adjacent to the boundary is less than the foam-wall friction, the foam slides over the first layers of bubbles adjacent to the boundary. The foam can move faster because the viscous friction between two layers of bubbles adjacent to the boundary is less than the foam-wall friction. The foam bubbles that are directly adjacent to the walls are observed in some cases to move slowly along the walls at a velocity significantly lower than that of the core bubbles. For slipping within internal layers of bubbles (internal plug flow), we observed that some of the remaining layer are thicker than one bubble from examining Fig. 9b/c closely near the injection hole. The remaining layer looks thicker near the injection hole than the other fingering areas.

As shown in Fig. 9c, air can flow through a narrow channel within the internal layers of bubbles in flow regime #3. In addition, air can split an interface leading to a bubble ‘poking’ its way along bubble interfaces with little displacement of the bulk of the foam as shown in Fig. 9d. For splitting of bubble interfaces, this is our hypothesis: from the observation of the sudden appearance and disappearance of a very thin channel in the thickness direction. The very thin channel in the thickness direction is marked with a yellow arrow in Fig. 9d in this document where the evolution of the bubbles are observed through time. When you look closely where the yellow arrow points, you will notice a small finger and a very blurry channel that connects the injection hole and the small finger. As shown in Fig. 9d, the blurry channel disappeared and closed, and the small finger stopped growing. Our hypothesis is derived from this observation of the very sudden opening and closing of a thin channel. Arif et al. observed a fracture-like brittle crack penetrating the foam that occurred by breaking films at much higher crack tip velocity of 10–20 m/s^23^. Yanagisawa et al. reported that a liquid film of foam bubble bursts with a velocity greater than 20 m/s^44^. In addition, finer, needle like fractures at the fingertips signal the onset of film ruptures in a radial Hele-Shaw cell^20^. In this study, the fingertip velocities are 0.5 m/s at 4.0 psi and 3.2 m/s at 10.0 psi. In addition, the fingering patterns show swelling shapes not the needle like fractures as seen by Arif et al. As a result, the dynamics of foam displacement flows seen in this study are dominated by a T1 process.

## Conclusions

The evolution of the viscous fingering interface for the immiscible radial displacement flows of air invading foam in Hele-Shaw cell has been examined. A commercial shaving foam, Gillette Foamy Regular, initially filled the cell. The foam was radially displaced by injecting air at constant pressures between 0.5 and 10.0 psi above atmosphere into the center of the Hele-Shaw cell. Three different flow regimes were identified. In a flow regime with low fingertip velocities at low air injection pressures, the foam is displaced slowly by plug flow with slip at the walls. In this regime, the air injection pressure is found to be proportional to the power of fingertip velocity with the exponent $\alpha =0.65$. This shows that the radial displacement flow with low velocities is in good agreement with the power law with the exponent $\alpha =2/3$ for bubbles moving in a cylindrical or polygonal capillary or a layer of bubbles slipping in a rectilinear Hele-Shaw cell. In a flow regime with high fingertip velocities at higher air injection pressures, the foam is displaced more rapidly leaving a layer of foam bubbles behind on the surface. The power law exponent is determined as $\alpha =0.36$ in this flow regime. Another flow regime was observed at 10.0 psi. At this high air injection pressure, some of the fingers developed as narrower channels where some of the air pockets formed in the foam became isolated as channels were ‘healed’ due to starvation (the air invaded other channels). The viscous fingering interfaces were quantitatively evaluated using the effective number of fingers, finger area density, and representative finger width density. The effective number of fingers and finger area density showed discontinuous or distinct patterns of change with respect to pressure in different flow regimes. On the other hand, the average finger width density continuously scaled with air injection pressure regardless of the flow regimes. The average finger width density scaling was determined as ${\overline{d} }_{w}\propto {P}^{-0.58}$, in agreement with the finger width scaling reported in the prior literature^27^.

## Data Availability

The datasets used and/or analysed during the current study available from the corresponding author on reasonable request.
